# Effect of Temperature on the Physical Salt Attack of Cement Mortars under Repeated Partial Immersion in Sodium Sulfate Solution

**DOI:** 10.3390/ma15186234

**Published:** 2022-09-08

**Authors:** Xing Jiang, Song Mu, Zheng Guo, Guangyan Liu

**Affiliations:** 1 School of Materials Science and Engineering, Southeast University, Nanjing 211189, China; 2 State Key Laboratory of High-Performance Civil Engineering Materials, Nanjing 211103, China; 3 Jiangsu Sobute New Materials Co., Ltd., Nanjing 211103, China

**Keywords:** physical salt attack, crystallization pressure, temperature, durability, cement-based materials

## Abstract

Physical salt attack (PSA) is one of the dominant durability issues of cement-based materials, where salt crystallization pressure is the driving force inducing damage. However, research on the temperature-related deterioration behavior of cement-based materials is limited. In this study, salt-contaminated cement mortars were rewetted at different temperatures. The assessment criteria were based on the visual appearance, weight evolution and size distribution of scaled materials, and the alterations in the microstructure were investigated by microscopy, thermal and mineralogical analyses. The results indicated that more severe damage developed at 5 °C than that at 20 °C due to the greater crystallization pressure caused by the conversion from thenardite (Na_2_SO_4_) to mirabilite (Na_2_SO_4_·10H_2_O) at the lower temperature. No damage was observed at 35 °C, since the repeated dissolution and re-crystallization of thenardite were harmless for the specimens. In addition, two distinct damage patterns were observed for PSA performed at 5 °C and 20 °C, namely, granular disintegration and contour scaling.

## 1. Introduction

Porous building materials such as stone, brick, masonry or concrete can be damaged due to pressure induced by the crystal growth of salts in their pores. While this issue was studied long ago in geomorphology and environmental science, it was often overlooked or misdiagnosed as chemical sulfate attack in concrete science. In recent years, physical salt attack on concrete by sodium sulfate has received increasing scientific interest. The American Concrete Institute (ACI) proposed the term “physical salt attack (PSA)” in order to distinguish it from the chemical sulfate attack caused by the chemical reaction of salts with cement hydration products or the main components of cement [1]. This mechanism is categorized as physical attack because chemical interactions between salts and materials are not involved in the damage process [2].

Long-term field investigations [3,4] found that the belowground portions of concrete half-buried in salt-laden soil were mostly in intact condition, whereas the aboveground portions of concrete exposed to air suffered various degrees of distress. It was concluded that PSA was the main cause of deterioration in the field-exposed concrete specimens. Similar cases of deterioration have been reported in the Gulf regions [5], Japan [6] and southern California [7]. However, the damage mechanism of cement-based materials partially immersed in sodium sulfate solution is still controversial. Because there is no standard method to assess the durability of cement-based materials to PSA, researchers often develop and apply their own test methods. Continuous partial immersion in sulfate solutions in a constant environment (20 °C and 60% relative humidity) is one of the most common test methods. Unfortunately, the field-like salt crystallization damage cannot be reproduced in such conditions. Some studies [8,9,10,11] concluded that the buildup of high concentrations of sulfate solutions at the evaporation zone resulting from wicking and evaporation could cause classical chemical sulfate attack. They hold the view that sulfate from sodium sulfate salts would react chemically with the hydration products to form gypsum and ettringite. In order to simulate the type of damage similar to that observed in the field, the continuous partial immersion of concrete in a sodium sulfate solution combined with thermal and humidity cycling was proposed [12,13,14,15]. As reported, this test procedure can reflect the damage caused by PSA well. Therefore, the discrepancy in the damage mechanism of the specimen partially immersed in sulfate solutions may be attributed to the different exposure conditions.

It is well known that the presence of a high percentage of micropores in cement-based materials increases the susceptibility to PSA. The increase in damage due to PSA was observed in cases of the replacement of cement by slag at ratios of 30% and 60% [14]. The field studies also found that the addition of slag to the binder at replacement ratios of 40% and 65% yielded a worse performance than plain concrete without slag [16]. Nehdi et al. [17] reported that the mass loss of the mixtures made with 8% weight replacement of Portland cement by silica fume was higher in comparison with cement-only mixtures. In a recent study, Ghafoori et al. [18] showed that the mortars containing 3 and 6% by weight cement replacement with nano-silica exhibited more severe scaling under PSA exposure in comparison to the control mixture. The addition and increase in cement replacement with other nanoparticles, such as nano-TiO_2_ [19] and nano-alumina [20], also provided a depressed resistance to PSA. The increased damage is the result of the excessive refinement of the pore structure due to the incorporation of supplementary cementitious materials (SCMs) or nanoparticles (nano-silica, nano-alumina and nano-TiO_2_), which would develop a high crystallization pressure, conforming to the salt crystallization theory proposed by Scherer [21]. On the other hand, concrete mixtures containing 20% cement replacement with Class F fly ash had a superior resistance to PSA [4,16,20]. A large number of macropores is believed to be present in these mixtures, which would provide additional space to accommodate the salts. Hence, these mixtures performed better than the mix made with only Portland cement. 

It appears that, during recent years, plenty of experimental studies have highlighted the relevance of pore-system characteristics to the decay of cement-based materials caused by PSA. Indeed, the damage caused by salt crystallization appears to be largely a function of the solution supersaturation ratio and the location of crystallization [22,23]. The solution’s physical properties (surface tension, viscosity and vapor pressure) and environmental conditions (temperature and relative humidity) have a critical effect on the dynamics of solution flow and evaporation, thus determining the degree of supersaturation and the depth of crystallization [24,25,26]. These key factors result in the damage level range, from very fine surface crumbling to severe progressive disintegration. In natural environments, PSA commonly occurs in arid regions [1], such as the southwestern United States; portions of southern Europe; coastal areas of Australia; much of the Middle East; and Northwestern China. Large differences in daily temperature are a distinguishing feature of these regions. For concrete half-buried in sulfate-rich soils, high crystallization pressure can be achieved as a result of the strong temperature dependence of the solubilities of the hydrated phase (mirabilite). Flatt et al. [27] reported that mass loss was observed after two cycles at 3 °C, while no damage was observed at 30 °C. Angeli et al. [26] also found that temperature is a key parameter controlling the extent of damage in the case of sodium sulfate decay. However, research on temperature-dependent salt crystallization attack of cement-based materials is limited.

This work aims to evaluate how the temperature affects the salt damage of cement mortars under an exposure condition that simulates PSA triggered by rising damp. In order to reduce the interference of chemical sulfate attack, cement mortar made with a high replacement of slag was used. The results from this study should provide a fundamental understanding of the role of temperature in the damage process.

## 2. Materials and Methods

### 2.1. Materials

In this study, P·I 42.5 Portland cement (PC) and ground-granulated blast furnace slag (GGBS) were used as the main components of the binder. The GGBS was used at 70% cement replacement by mass to reduce the interference of chemical sulfate attack. The chemical composition and physical properties of the cement and GGBS are given in Table 1. The chemical compositions were determined by X-ray fluorescence (XRF). The sodium sulfate solutions were prepared from anhydrous sodium sulfate (Na_2_SO_4_, >99% purity) at concentrations of 5% and 13% by mass. Sodium sulfate was selected because it is common in saline soils and has a long history of being linked to physical attack in concrete structures [1].

### 2.2. Specimen Preparation

For mortar mixtures, China ISO standard sand manufactured according to EN196-1 was used. In order to accelerate the deterioration process, a large water-to-binder ratio of 0.55 was used to prepare the mortar prisms. The binder–aggregate ratio was kept constant at 1:3 by mass. Mortar prisms were cast in 40 mm × 40 mm × 160 mm steel molds. The specimens were cured in sealed conditions for 24 h at room temperature (20 ± 2 °C) and then demolded and cured at standard conditions (20 ± 2 °C and 98% RH) until 28 days of age.

### 2.3. PSA Exposure

#### 2.3.1. Pre-Conditioning of Specimens

**Figure 1:**
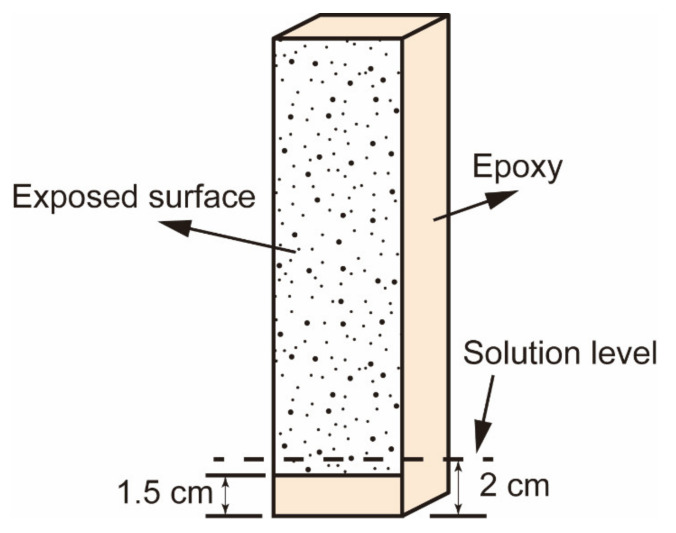
Schematic representation of the specimen with epoxy coating.

After the standard curing for 28 days, the mortar specimens were left in the laboratory (20 ± 2 °C and 60% RH) for 48 h to eliminate the moisture from the surfaces of the specimens. There are two factors that will lead to the assessment of damage evolution being biased when mortar prisms are partially immersed in a salt solution. On the one hand, the mass increase due to the repeated salt contamination of submerged portions complicates the mass change of the specimen caused by the surface scaling of air-exposed portions. On the other hand, the edges of the specimen might suffer more severe damage than the surfaces due to two-dimensional or three-dimensional exposure to a salt solution. In order to extract more relevant and objective information on the evolution of salt decay, one-dimensional exposure to physical salt attack was proposed in this study. For that, a resin layer was applied to the part of the exposed surface and the remaining sides, as shown in Figure 1. When the specimens were partially immersed in sulfate solutions, the solution level was kept slightly higher than the upper side of the epoxy covered on the exposed surface so that the salt solutions could be drawn into the drying portion of the specimen through capillary action. 

#### 2.3.2. Exposure Conditions

**Figure 2:**
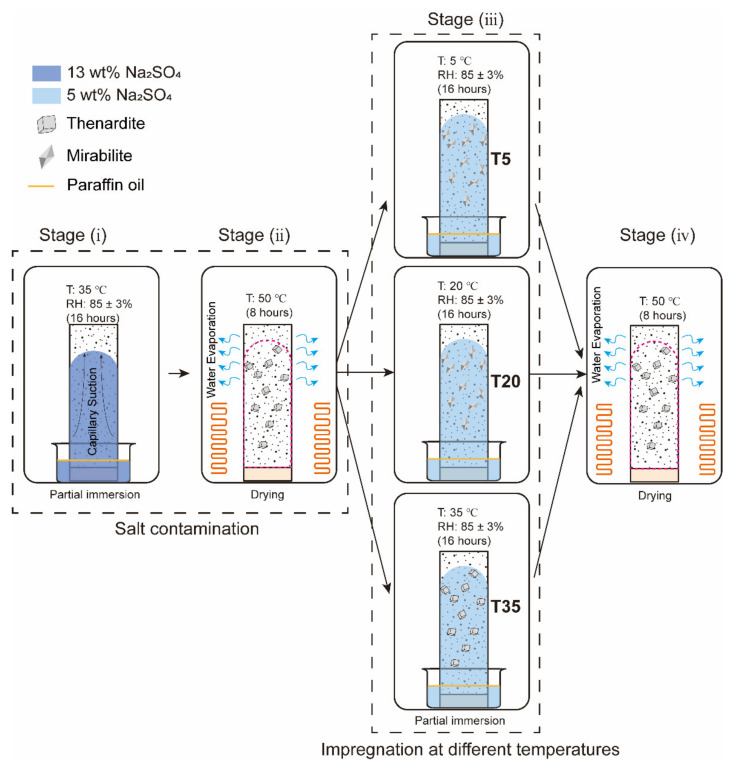
Schematic diagram showing the PSA exposure of cement mortar prisms. At a temperature higher than 32.4 °C, thenardite is the only thermodynamically stable phase [33].

**Figure 3:**
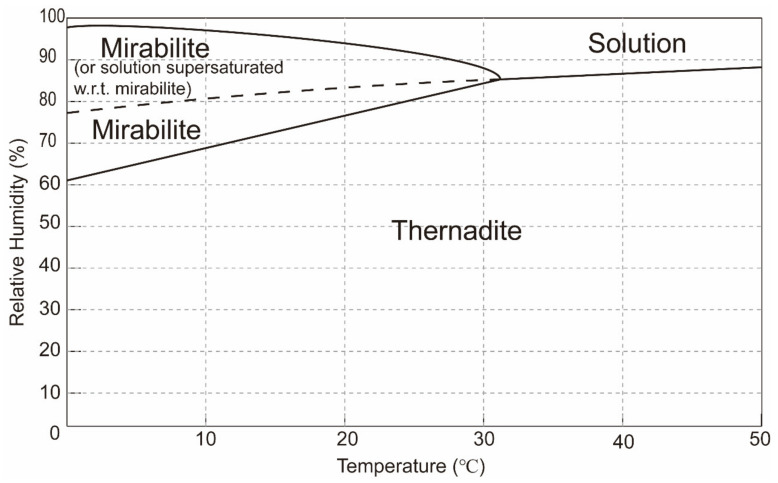
Phase diagram for sodium sulfate. The continuous lines indicate the boundaries of the stable phases. The discontinuous line corresponds to a solution in metastable equilibrium with respect to thenardite and supersaturated with respect to mirabilite. The bi-directional arrow indicates the predominate exposure regime used by researchers. (After Flatt [29], with permission from Elsevier.).

Nine specimens were subjected to accelerated aging tests adapted from the exposure condition proposed by Nehdi et al. [17]. Eleven full cycles have been performed, where each cycle (48 h) consisted of four consecutive stages: partial immersion at 35 °C, drying, partial immersion at different temperatures (5 °C, 20 °C and 35 °C) and drying, as shown in Figure 2. The timing for each stage is designed based on Bassouni and Rahman [14]. More details are as follows:

(i) 16 h partial immersion in 13 wt% sodium sulfate solution at 35 °C. The high-concentration sodium sulfate solution was selected to accelerate the salt accumulation in the drying portion of the specimen.
(ii) All specimens were removed from the container and placed in an oven for 8 h. The temperature of the oven was set to 50 °C to avoid the alteration of the pore structure [28]. Thenardite (Na_2_SO_4_) was expected to be the stable phase in this stage based on the phase diagram of sodium sulfate [29], as shown in Figure 3.
(iii) Every three salt-contaminated specimens were partially immersed in 5 wt% sodium sulfate solutions at 5 °C, 20 °C and 35 °C for 16 h, separately marked as specimens T5, T20 and T35. The 5 wt% sodium sulfate solution was close to the concentration suggested by ASTM C1012 [30]. At low temperatures (5 °C and 20 °C), as in the ASTM [31] and EN 12370 [32] tests, the crystallization is driven by the dissolution of thenardite that is formed during stage (ii) and the formation of a solution supersaturated with mirabilite (Na_2_SO_4_·10H_2_O). At 35 °C, above the thenardite-mirabilite transition temperature of 32.4 °C, no supersaturated solution forms during this stage.
(iv) All specimens were subjected to drying under 50 °C for 8 h.

The reference specimens were not exposed to PSA and sealed with polyethylene film (henceforth abbreviated as Ref). The ambient humidity of 85 ± 3% RH was selected to prevent the salt efflorescence from developing in the drying portion of the specimen. During the partial immersion stage, ambient environments were created within desiccators whose relative humidity and temperature were controlled. The RH was maintained at 85 ± 3% at varied temperatures using saturated KCl solution. A film of paraffin oil was placed on top of the solution to promote the migration of the solution through the pore system of the cement mortar and to minimize solution evaporation. The solutions were replenished after every cycle to maintain the solution level at the height of 2 cm.

### 2.4. Measurement

#### 2.4.1. Marco-Structural Monitoring

Mortar prisms were visually monitored at the end of the test. After each cycle, the scaled materials were collected, washed, dried and weighed. The specimens were cleaned with a nylon brush to remove debris from the surfaces. The debris in the solutions that fell from the specimens was also collected. To remove the salts from the debris, they were immersed in 50 °C de-ionized water to prevent damage during cleaning. Subsequently, the debris was collected using vacuum filtration and dried in an oven at 105 °C for 24 h. The debris was classified into three categories: <0.075 mm, 0.075–2.36 mm and >2.36 mm. Each category was weighed and expressed as the percentage of the debris weight. The cumulative scaled materials were calculated as the sum of the average weight of the debris from each specimen. At the end of each cycle, the weight of the specimens was obtained using a balance with an accuracy of 0.01 g. The evolution of specimen weights was normalized according to Equation (1):

(1) $$normalized\;weight\;at\;\left(i\right)\;cycle\left(s\right)=\frac{M_{i}}{M_{0}}$$

where *i* is the number of cycle(s), *M*_0_ is the weight of each specimen before PSA exposure and *M_i_* is the weight of each specimen after *i* cycle(s).

#### 2.4.2. Characterization of Mineralogical and Microstructural Alteration

**Figure 4:**
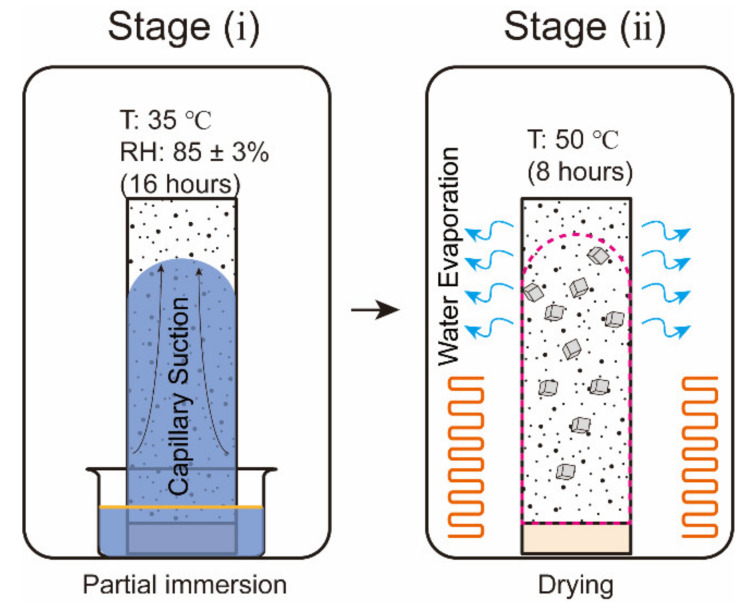
Schematic representation of the salt contamination procedure.

Another nine mortar prisms were subjected to cycles of the salt contamination procedure (stages (i) and (ii)) to obtain the salt-contaminated specimens, as shown in Figure 4. These salt-contaminated specimens are labeled SC-N, where SC denotes salt contamination and N denotes the number of cycles.

**Figure 5:**
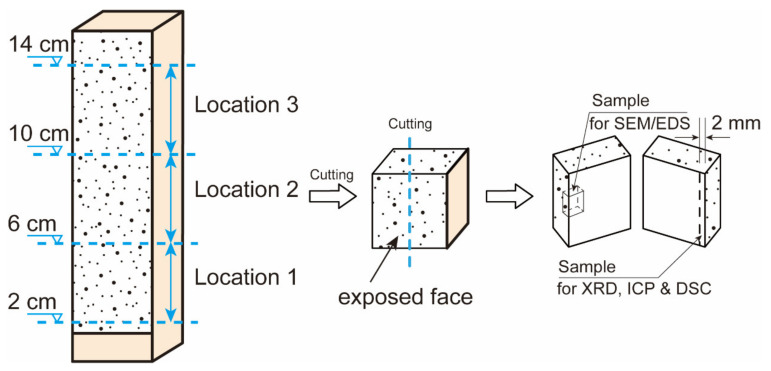
Sample collection and preparation for microstructural analysis.

Three cubes (Locations 1, 2 and 3) were cut from the salt-contaminated specimen using a saw cooled with isopropanol, as shown in Figure 5. The powder samples were collected from the surface (0–2 mm from the drying surface) by grinding. Before profile grinding, the epoxy on the specimen was removed. Thereafter, the powder was dried and passed through a 75 μm sieve. X-ray diffraction (XRD, D8 Advance, Bruker, Billerica, MA, USA) with Cu-Kα radiation generated at 40 mA and 40 kV was carried out on the fine powder. Differential scanning calorimetry (DSC) was conducted with a uniform heating rate of 10 °C/min from room temperature (20 ± 1 °C) to 1000 °C in a nitrogen environment. Approximately 1 g of the powders was used to quantify the water-soluble sulfur and sodium contents using an induced coupled plasma optical emission spectrometer (ICP) from Spectro (Kleve, Germany). After drying for 24 h at 105 °C, the powder was weighed. Then, the dried powder was dissolved in 10 mL of deionized water and filtrated after 24 h. The contents of sulfur and sodium were expressed relative to the dry mortar mass. The thin sections used for SEM/EDS were extracted from the surface of specimens at Locations 1, 2 and 3, respectively. The fracture surfaces were polished and coated with gold to enhance the conductivity. A FEI Quanta 3D scanning electron microscope (SEM, Hillsborough, CA, USA) combined with an Oxford XMax silicon drift energy dispersive spectrometer (EDS, Oxford, UK) were used to investigate the distribution of salts in these samples. Observations were carried out in high vacuum mode with an accelerating voltage of 15 kV.

## 3. Results

### 3.1. Macroscale Observation

#### 3.1.1. Visual Appearance

**Figure 6:**
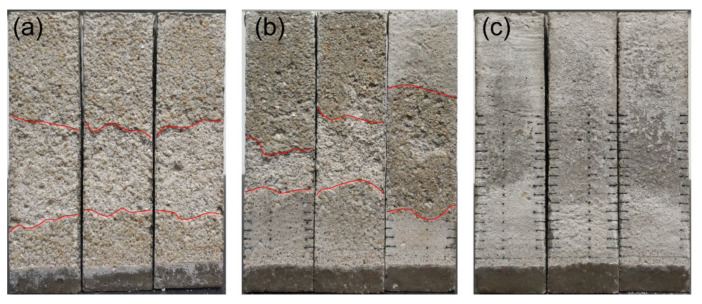
Visual appearance of the specimens at the end of the PSA exposure test: (**a**) specimen T5; (**b**) specimen T20; (**c**) specimen T35.

It can be observed that the specimens T5 and T20 suffered from deterioration, whereas the specimen T35 remained intact throughout the cycles of the salt crystallization test, as shown in Figure 6. Moreover, damage only occurred during the stage at which the salt-contaminated specimens were partially immersed in 5% Na_2_SO_4_ solutions (stage (iii)). For specimen T5, the damage occurred during the first cycle. The decay pattern manifested mainly as granular disintegration. For specimen T20, the deterioration started to appear during the fifth cycle. During the 5–7 cycles, the decay pattern was similar to that of specimen T5. From the eighth cycle, flaking started to appear and became the main type of decay in the following cycles. In addition, the specimens exhibited higher surface scaling at Location 2, regardless of the temperature, compared with that at Locations 1 and 3.

#### 3.1.2. Weight Evolution

**Figure 7:**
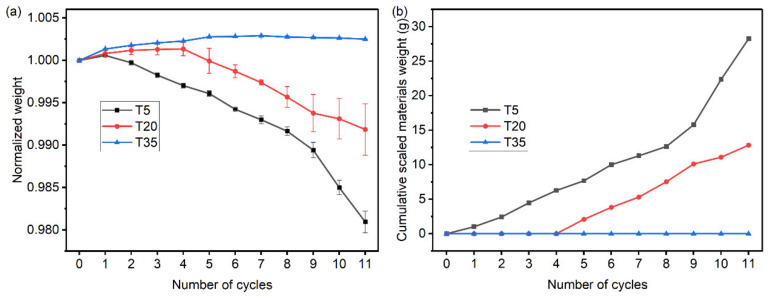
(**a**) Weight evolution of the specimens in the dry state. (**b**) Cumulative scaled materials weight due to surface scaling.

Figure 7a presents the normalized weight of all the tested specimens as a function of cycles. For specimen T35, the specimens gradually increased in mass until the fifth cycle. After that, they were almost kept at a constant weight. Since specimen T35 was kept in good condition, the increase in mass was caused by the precipitation of anhydrous Na_2_SO_4_ in the porous network of the specimen due to the continued absorption and evaporation of Na_2_SO_4_ solutions. For specimens T5 and T20, the evolution of dry specimen weights exhibited a gain at the beginning of the exposure and then decreased when the damage started. The mass growth of specimen T20 was greater than that of specimen T5, indicating that the former needed more salt to induce the damage. In addition, the lower temperatures led to more weight loss. At the end of exposure, the normalized weight of cement mortars reached 0.980 and 0.991 for specimen T5 and specimen T20, respectively. Because the changes in mass are the result of the competition between salt precipitation and surface scaling during a full salt crystallization cycle, monitoring the mass change of specimens has its limitations in determining the occurrence of damage. For instance, specimen T5 already showed surface scaling after the first cycle based on the visual appearance, but the mass of the specimen in the dry state increased. In order to extract more relevant and precise information on the PSA, the desalinated scaled materials were collected to track the progression of damage (Figure 7b). It should be mentioned that collecting the scaled material from specimens provides a pretty good insight into the development of damage, since it can monitor the signal of damage onset as the visual evolution did.

#### 3.1.3. Size Distribution of Scaled Materials

**Figure 8:**
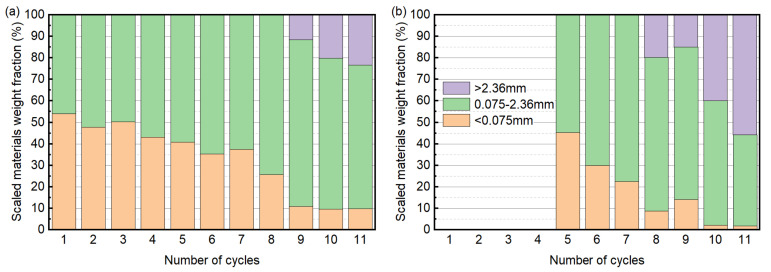
Weight of different sizes of the fragments, expressed as a percentage of the debris weight after each cycle: (**a**) specimen T5; (**b**) specimen T20.

To gain the quantitative information on the type of damage, the scaled materials were categorized according to the size of the fragments, as shown in Figure 8. For specimen T5, the scaled materials mainly consisted of particles less than 0.075 mm and in the range of 0.075–2.36 mm, suggesting that granular disintegration was the main type of decay. In addition, the weight percentage of the particles less than 0.075 mm decreased with cycles. This can be attributed to the scaling of the hardened cement paste layer that covered the specimen. After the complete detachment of the paste layer, the ratio of particles less than 0.075 mm and particles in the range of 0.075–2.36 mm could be relatively stable. From the nineth cycle, particles larger than 2.36 mm started to appear, and the weight percentage of these particles increased with cycles. It can be noted that the trend of normalized weight change is consistent with that of the weight percentage of particles with a large size in the sense that a high mass loss corresponds to a large percentage of particles larger than 2.36 mm. For specimen T20, the particles larger than 2.36 mm became the dominating component, and the weight percentage of particles less than 0.075 mm was generally low. This suggests that the damage pattern is mainly in the form of contour scaling. At the end of the test, the weight percentage of the particles larger than 2.36 mm exceeded 50%, while only 1.8% of particles less than 0.075 mm were produced. Furthermore, the appearance of large particles disperses the weight evolution aggressively, and the impact is amplified when there are more particles larger than 2.36 mm produced in debris.

### 3.2. Alteration in Microscopic Properties

Due to damage in the form of surface scaling, the damaged specimens cannot provide full information in terms of the alteration of microstructural properties. Thus, the specimens subjected to cycles of salt contamination (stages (i) and (ii)) were used for microanalysis. The salt contamination was performed for 1, 5 and 11 cycles, respectively, to obtain the amount and distribution of salt in the specimens before the damage onset, which were separately marked as SC-1, SC-5 and SC-11.

#### 3.2.1. Mineral Compositions of Salt-Contaminated Specimens

**Figure 9:**
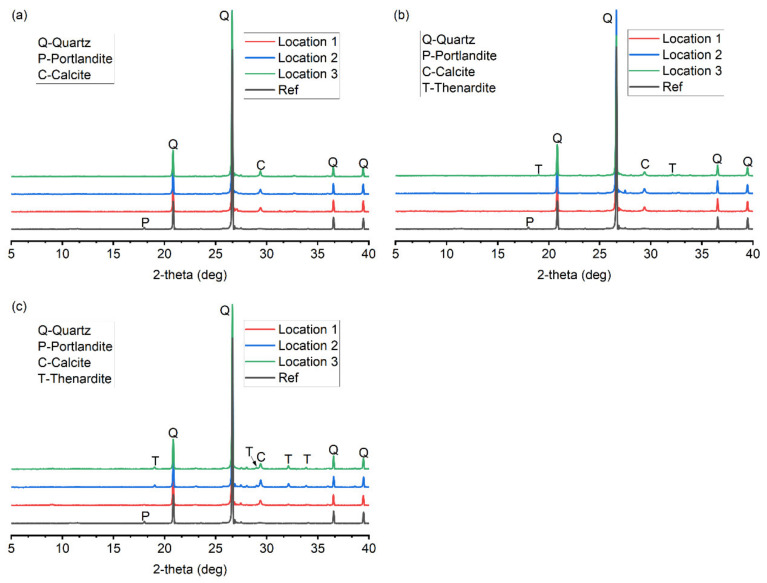
XRD patterns of salt-contaminated specimens: (**a**) SC-1; (**b**) SC-5; (**c**) SC-11.

Figure 9 shows the XRD analysis of the reference and salt-contaminated specimens. For the reference sample, some traces of portlandite were found. This is the result of the GGBS chemically reacting with portlandite and water to produce secondary C-S-H and the dilution effect. Calcite was detected in all the salt-contaminated specimens, indicating that the outer surface (0–2 mm) of the drying portion had been carbonated. Specimen T5 showed surface scaling after one cycle of PSA exposure, indicating the presence of thenardite. However, thenardite cannot be detected by XRD in the drying portion of SC-1. This can be explained by the large amount of quartz in the powder samples, because the quartz peaks are too high and may mask the characteristic peaks of thenardite. Similarly, thenardite can be identified only at Location 3 of SC-5. The identification of thenardite in SC-5 shows the buildup of thenardite with the cycles of salt contamination. It substantiates that more thenardite is needed to cause damage at 20 °C. Regarding SC-11, thenardite can be detected at Locations 1, 2 and 3. This means that a large amount of thenardite had been precipitated in the drying portion of the specimen after 11 cycles of salt contamination. Nevertheless, no damage was observed on the specimen T35. This study confirms that the crystallization of thenardite is almost harmless for cement mortars because there is no phase transition between thenardite and mirabilite at 35 °C.

**Figure 10:**
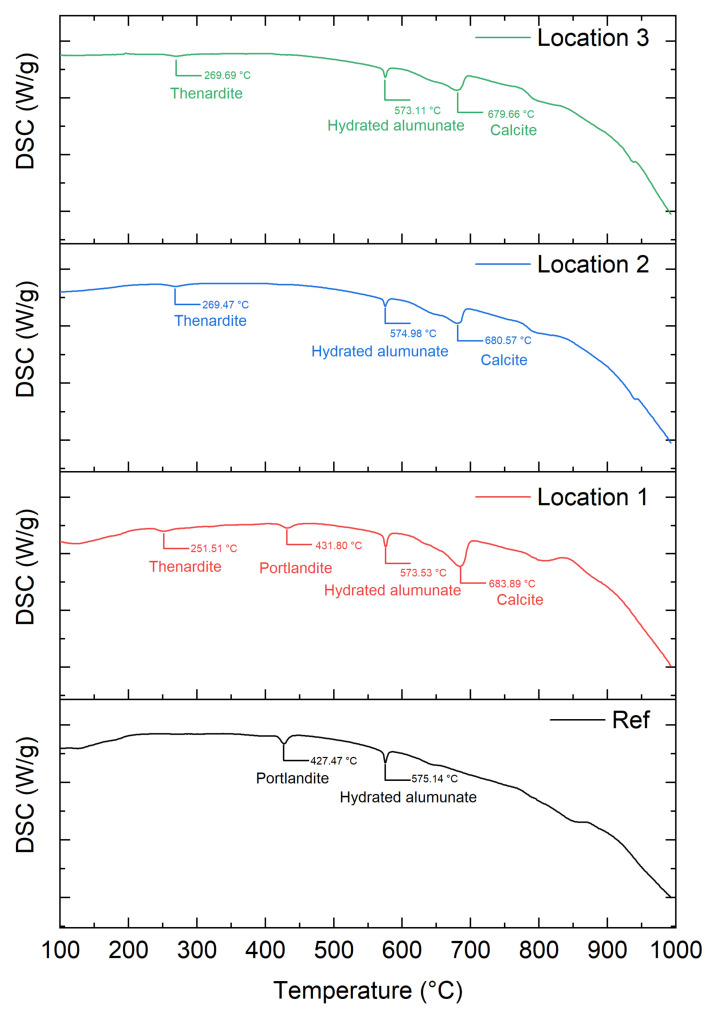
DSC data of the reference sample and the specimen SC-11.

To complement the analysis from the XRD, the mineral compositions of SC-11 were analyzed by DSC (Figure 10). In the DSC curve, the peak intensity in the case of the reference sample was remarkable at 427 and 575 °C, which indicates the decomposition of portlandite and hydrated aluminate [34], respectively. For the salt-contaminated samples, the DSC curve showed an endothermic peak in the range of 670–690 °C corresponding to the decomposition of calcite. At Location 1, there was a minor peak attributed to portlandite dehydration (430 °C), indicating that the portlandite was not completely consumed by the formation of calcite. It is possible that Location 1 is covered with the solution, which hinders the ingress of CO_2_. The disappearance of this peak at Locations 2 and 3 showed that the carbonation in these regions was more significant. The endothermic peak that appeared in the range of 250–270 °C was due to the phase transformation of sodium sulfate crystals [35], which is consistent with XRD in substantiating that salt crystallization is the main mechanism for the surface scaling of specimens.

#### 3.2.2. Distribution of Salt in the Specimen

The damage in the form of surface scaling and the presence of thenardite substantiate that the salt crystallization is responsible for the damage in the drying portion of the specimen. The visual appearance showed that the most severe damage was localized at Location 2. This feature should be related to the distribution of sodium sulfate crystals in the drying portion of the specimen.

**Figure 11:**
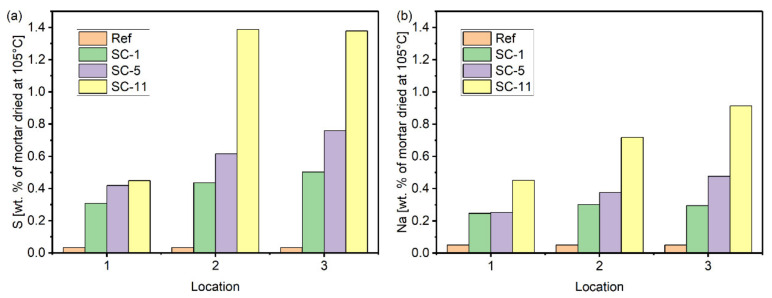
Water-soluble sulfur (**a**) and sodium (**b**) content determined by ICP for the reference and salt-contaminated specimens.

The measurement of the water-soluble sulfur and sodium content is given in Figure 11. The results showed that more sodium and sulfur accumulated in the drying portion of the specimen as the number of cycles increased. The amount of sulfur and sodium at Location 1 was significantly lower than that at Locations 2 and 3, suggesting that more thenardite was accumulated in the regions further from the solution level, conforming to the XRD trends. The distribution of sodium sulfate crystals along the longitudinal profile can be related to the capillary rise and the evaporation of the salt solution. When the specimens are partially immersed in sodium sulfate solutions, the solution will be drawn into the drying portions of the specimen through capillary pressure. The flux from the capillary rise decreases as the height increases based on Darcy’s law [21], whereas the rate of evaporation is approximately constant. At Location 1, the rate of capillary rise is greater compared with the rate of evaporation, so there is a liquid film on the surface of the specimens. Therefore, salt is not easily enriched at Location 1. As the height increases, salt crystallization could take place when the rate of evaporation exceeds the rate of capillary rise. Thus, Locations 2 and 3 were manifested as larger amounts of crystallizing salt.

**Figure 12:**
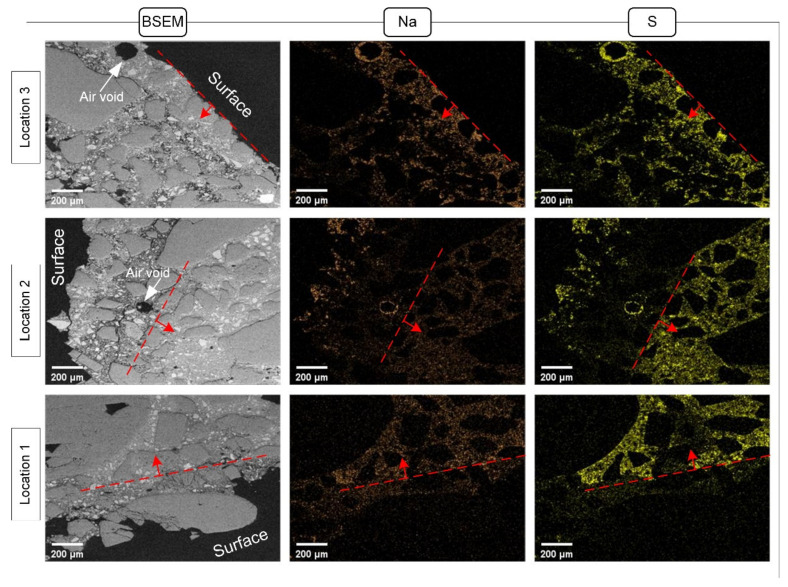
Elemental maps of the drying portion of the specimens after 11 cycles of salt contamination. The red dashed lines show the front of crystallization.

Figure 12 shows the BSE images and the elemental maps for the specimen subjected to 11 cycles of salt contamination. The results consist of maps of the relative concentrations of sodium and sulfur, which reflect the distribution of thenardite at the outer section of the drying portion. It can be found that thenardite crystals precipitated both in the air voids and in the pores of the mortar mixtures. The thin slices extracted from Locations 1 and 2 showed that salt accumulation mainly occurred further from the surface, while salt accumulation close to the surface was detected at Location 3. The distribution of salt in the cross-sectional direction can be explained by the competition between the capillary rise and evaporation. While the specimens were partially immersed in the salt solution, salt could crystallize out from the evaporation solution at Location 3 due to evaporation being dominant. Therefore, the evaporation front remained at the surface for the salt solution to reach and crystallize on it. However, salt crystallization was less likely to occur at Locations 1 and 2 due to the predominance of capillary rise. Salt crystallization in these two locations tends to occur in the subsequent drying stage. In such a case, the drying front entered the specimen rapidly, and salt crystallization occurred away from the surface of the specimen.

## 4. Discussion

### 4.1. The Role of Salt Contamination

**Figure 13:**
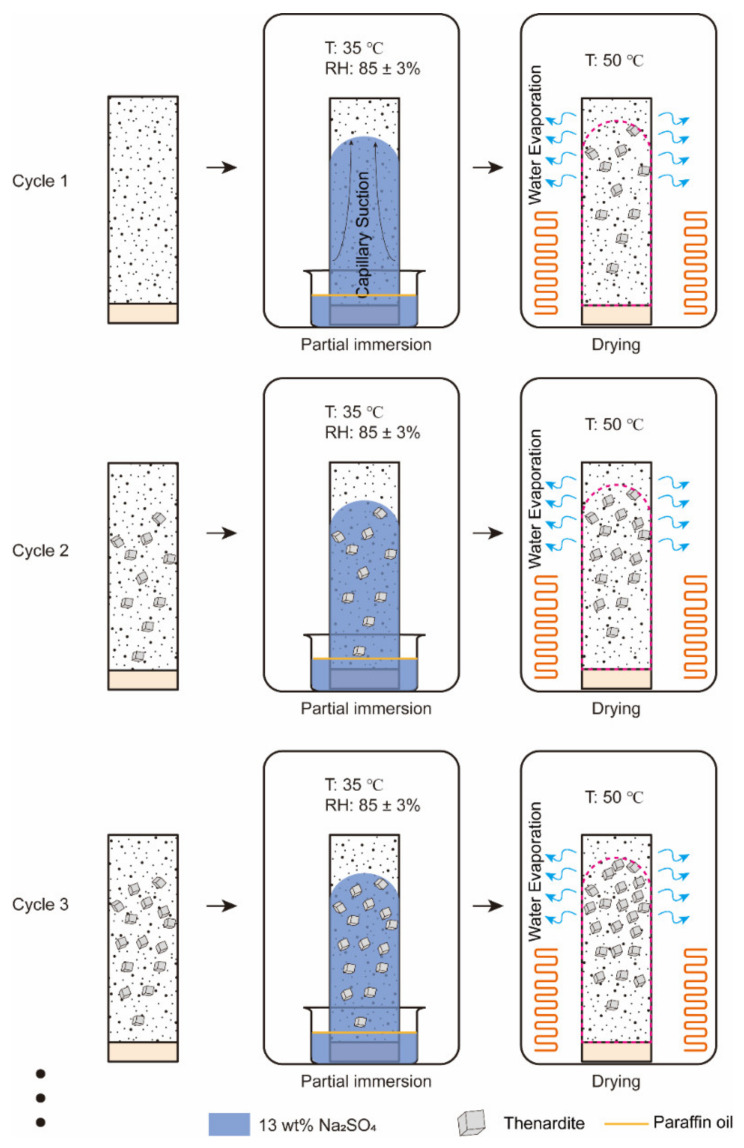
Schematic presentation of the salt accumulation process in the cement mortar.

The salt contamination process was introduced in the accelerated salt crystallization test with the aim of accelerating the accumulation of salt in the specimen under conditions that do not cause damage to the specimen. A study conducted by Angeli et al. [26] showed that progressive mass gain without surface scaling was observed in a Lutetian limestone subjected to sulfate solutions with different concentrations at 50 °C. In addition, the increased mass is proportional to the concentration of the solution. Another PSA study by Tsui [36] also found that no degradation occurs at 50 °C. In this study, the specimens subjected to the cycles of salt contamination would undergo the repeated crystallization of thenardite, but no damage was observed, which is consistent with previous studies [26,36]. On the other hand, the thenardite content that increased with cycles was investigated by ICP and XRD. A proposed mechanism for salt accumulation is shown in Figure 13. When the salt-contaminated specimens were partially immersed in Na_2_SO_4_ solution again at 35 °C, the salt solution rose to salt-contaminated regions through capillary action, leading to the dissolution of thenardite instead of the phase change between thenardite and mirabilite. Thus, the concentration of sodium sulfate solution in the evaporation zone became higher. Consequently, more thenardite will form in the evaporation zone. This is also reflected in the increase in the mass of the specimen with cycles before the occurrence of surface scaling (Figure 7a). The PSA performed at a temperature above the thenardite-mirabilite transition temperature will cause salt accumulation instead of surface scaling.

Some studies [37,38] concluded that chemical sulfate attack is responsible for the damage occurring in the evaporation zone because a large amount of ettringite and gypsum were detected instead of thenardite. The absence of thenardite can be attributed to the continuous partial immersion performed in these studies. Generally, the surface of the evaporation zone is permanently covered with a layer of saturated salt solution on which mirabilite is formed as a transparent crust [39,40]. Thick salt efflorescence will develop on the transparent crust in an environment that promotes the formation of thenardite. In such a case, the evaporation zone is inclined to undergo a chemical sulfate attack. Thus, the continuous partial immersion of the specimen in the Na_2_SO_4_ solution is not a good choice for the accumulation of salt in the specimen. By comparison, partial immersion in a humid environment and drying the entire specimen can avoid the development of efflorescence on the surfaces of the specimen and promote the accumulation of crystals within the specimen. A specific salt amount can be obtained using this salt contamination procedure to establish a relationship between the salt contents and the damage onset of the specimen.

The concrete structures that are half-buried in salt-laden soil are actually exposed to cycles of wetting and drying due to the periodicity of precipitation. The accumulation of salt is achieved in this process. Therefore, although the salt contents in soils are low, PSA could be triggered when the numbers of wetting and drying for salt accumulation are sufficient [4]. In laboratory tests, a very high concentration of sodium sulfate solution was commonly selected to accelerate the damage process. It is a good choice to facilitate the salt accumulation within a reasonable time, but the dissolution-crystallization events achieved by rewetting the specimens with a high concentration of a salt solution are not common in practice. Therefore, the salt contamination and the dissolution-crystallization should not coincide, as they did in this study.

### 4.2. The Role of Temperature

According to Coussy [41], the local crystallization stress (Δp) can be related to the macroscopic tensile stress (σ) by

(2) $$\sigma =\Delta pbS_{C}$$

where b is the Biot coefficient and $S_{C}$ is the volume fraction of the porous network filled with sodium sulfate crystals. Equation (2) shows that the amount of salt accumulated and the crystallization pressure play important roles in determining the damage onset.

**Figure 14:**
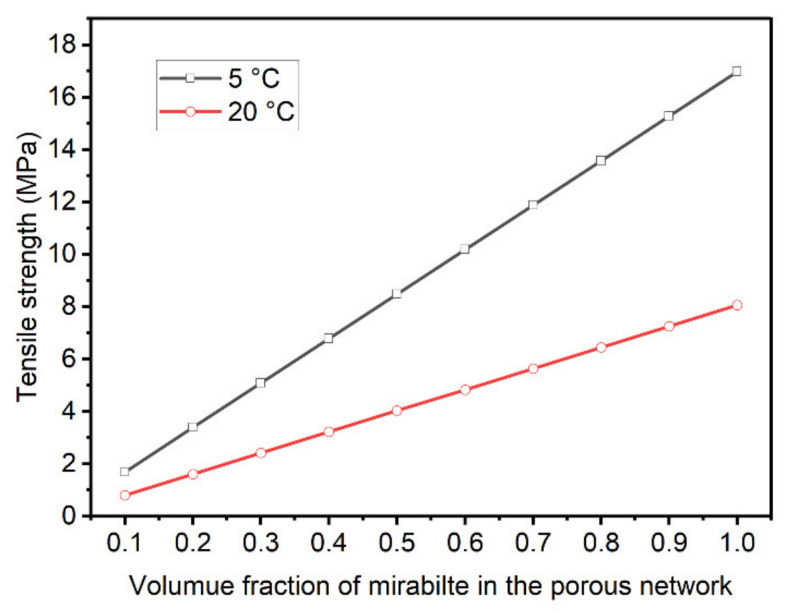
Evolution of the predicted macroscopic tensile strength.

The experimental results showed that the specimens re-wetting at a lower temperature required fewer salt crystallization cycles for damage to occur. The result can be attributed to the level of supersaturation achieved during re-wetting and the amount of salt precipitated in the porous network of specimens. After the salt contamination procedure, thenardite was the stable phase that was deposited in specimens in accordance with the microstructural analyses. When re-wetting occurred at 5 °C and 20 °C, crystallization in this case is believed to be driven by the dissolution of thenardite and the formation of a solution highly supersaturated with respect to mirabilite. The supersaturation and, therefore, crystallization pressure increase with the decrease in temperature because of the strong temperature dependence of the solubility of mirabilite [29]. According to Pitzer’s interaction model [42], the crystallization pressure for mirabilite forming in a solution saturated with respect to thenardite at the temperatures of 5 and 20 °C is 29.26 and 13.91 MPa, respectively. In this study, the Biot coefficient is assumed to be 0.58 for the cement mortar based on the study by Skoczylas [43], which should be carefully validated in the future. Thus, the macroscopic tensile stress can be estimated, as shown in Figure 14. For a given $S_{C}$, the tensile strength generated by salt crystallization is observed to increase with the decrease in temperature. This is why the specimen T5 suffered from deterioration at an earlier time, although a small amount of salt was deposited in the pore network (Figure 11). For the specimen T20, a higher degree of pore space filling and, thus, more salt contamination cycles were needed to cause damage. For the specimen T35, the specimens would undergo the dissolution of thenardite instead of the conversion from thenardite to mirabilite, and no supersaturation solution would form during the re-wetting stage. The re-crystallization of thenardite only occurred during the drying at 50 °C. In this case, the efficiency of pore space filling is limited due to the molar volume of thenardite being a quarter of that of mirabilite. Our results are generally consistent with those of Flatt et al. [27], who reported that the Portland limestone exhibited a fast mass loss at low temperatures. Additionally, Angeli et al. [26] also showed that the most damaged samples are those at 5 °C compared to those at room temperature and at 50 °C. The results from the current study and those of previous studies suggest that the damage is caused by the conversion from thenardite to mirabilite, and the damage will be elevated when the salt-laden specimens are rewetted with a colder sulfate solution. 

An interesting thing to notice is that the most severe damage did not appear at Location 3, where the largest amount of thenardite was deposited. This may be the result of pore clogging caused by the formation of mirabilite. The conversion of thenardite to mirabilite is associated with 314% volumetric expansion during the partial immersion of specimens in 5% sodium sulfate solutions, which could cause pore clogging, preventing the salt solutions from reaching Location 3. It is reasonable to infer that the location in which the conversion from thenardite to mirabilite takes place, rather than the amount of salts accumulating in the specimens, appears to be more relevant in the development of damage.

Temperature also has an influence on the damage pattern of specimens. According to Steiger [44,45], the minimum pore radius for the growth of mirabilite decreases with supersaturation. This means that the volume of percolated porosity in which mirabilite can grow increases with supersaturation [46]. Therefore, the specimen T5 showed damage manifestation as granular disintegration as a result of salt crystallization in smaller pores. For the specimen T20, stress fields were generated by the growth of mirabilite in larger pores. In this case, the detached parts seem to be localized by flaws in the specimens [26]. Thus, a considerable number of particles larger than 2.36 mm were formed in the debris. This observation agrees well with our previous work [47].

## 5. Conclusions

This research investigated the influence of different temperatures (5 °C, 20 °C and 35 °C) on the damage evolution of cement mortars that repeated partial immersion in sodium sulfate. The physical salt attack (PSA) exposure conditions simulate the condition in which materials are periodically exposed to rising damp, which is a common condition in practice. Based on the experimental results, the following conclusions can be drawn:

- Damage was observed by the scaling of the outer surface of the cement mortar at the locations above the solution level. The surface scaling mainly developed in the middle zone of the drying portion of the specimen since more thenardite was accumulated in these regions.
- Scaling occurred during the formation of mirabilite at temperatures of 5 °C and 20 °C. No damage was observed when the PSA exposure was performed at 35 °C, above the thenardite-mirabilite transition temperature, showing that the crystallization of thenardite was almost not dangerous to the cement mortar.
- PSA damage was more significant at 5 °C than at 20 °C, and this can be attributed to the higher degree of supersaturation achieved during the conversion from thenardite to mirabilite at the lower temperature. At the end of the exposure, the specimens T5 and T20 lost 2.0% and 0.9% of their initial weight, respectively. The damage pattern also varied with the temperature. At 5 °C, granular disintegration occurred, and the scaled materials contained 75–100% particles smaller than 2.36 mm in size. At 20 °C, contour scaling resulted in the loss of 20–50% of flake with a diameter greater than 2.36 mm.
- This study qualitatively illustrates the effect of salt accumulation on the development of damage. Further research is needed to quantify the relationship between the pore filling volume and the onset of damage under different temperatures.

## Figures and Tables

**Table 1:** Chemical composition and physical properties of PC and GGBS.

	PC	GGBS
Chemical composition		
SiO_2_ (%)	19.10	30.10
Al_2_O_3_ (%)	4.61	14.50
Fe_2_O_3_ (%)	3.10	0.80
CaO (%)	62.75	40.10
MgO (%)	1.87	8.19
SO_3_ (%)	2.62	0.38
Na_2_O (%)	0.17	0.49
K_2_O (%)	0.42	0.51
Physical properties		
Specific surface area (m^2^/kg)	342	373
Specific gravity (g/cm^3^)	3.07	2.85
